# Realities of opioid and gabapentinoid deprescribing in socioeconomically disadvantaged communities: a qualitative evaluation

**DOI:** 10.3399/BJGPO.2024.0160

**Published:** 2025-01-15

**Authors:** Charlotte L Parbery-Clark, Jennie Sofia Portice, Sarah Sowden

**Affiliations:** 1 Population Health Sciences Institute, Faculty of Medical Sciences, Newcastle University, Newcastle, UK

**Keywords:** general practice, deprescribing opioids and gabapentinoids, deprescriptions, analgesics, opioid, qualitative research

## Abstract

**Background:**

Opioid and gabapentinoid prescribing has increased substantially in recent years despite having limited effectiveness in treating chronic primary pain. This is concerning, with the prescribing rates and adverse effects of these medications being higher in more socioeconomically disadvantaged groups. Guidance for prescribing and deprescribing these medications exists but the understanding of how deprescribing is operationalised, especially in areas of socioeconomic disadvantage, is limited.

**Aim:**

To explore primary healthcare professionals' views and experiences of designing and implementing an intervention to reduce opioid and gabapentinoid prescribing.

**Design & setting:**

A qualitative evaluation, using participant observation and semi-structured interviews with primary healthcare professionals, working in practices serving areas of substantial socioeconomic disadvantage in the North East of England.

**Method:**

Interviewees were purposively recruited with subsequent snowballing with participant observation of the peer-support meetings. Interview transcripts and notes from the participant observation were inductively coded and thematically analysed.

**Result:**

Thirteen healthcare professionals from five practices were interviewed. Person-centred care with shared decision-making was strived for, which was time-consuming owing to the complexity of the problem and patients. Where shared decision-making was not possible, owing to patient refusal or non-engagement, risk was used to determine the appropriate action. This work involved an emotional toll on staff and patients, but was at times conversely easier and more rewarding than expected. Ultimately, demedicalising pain with a culture change is required to ensure patients are not prescribed these medications for inappropriate reasons or doses.

**Conclusion:**

This study demonstrates key operational aspects to consider when undertaking opioid and gabapentinoid deprescribing in primary care, such as funding dedicated time to enable deprescribing.

## How this fits in

There is increasing evidence that opioids and gabapentinoids have limited effectiveness in the treatment of chronic primary pain (including chronic non-cancer pain) with the risks outweighing the benefits. This study found that funding dedicated time enabled general practices, serving areas of deep socioeconomic deprivation, the flexibility to design and implement an opioid or gabapentinoid deprescribing intervention using person-centred care principles. The work was emotive and included considering risk to make decisions and prioritise the work. Ultimately, a culture change is required, in not only primary care but also the wider healthcare system, to ensure that patients are not on inappropriate doses of these medications for inappropriate reasons.

## Introduction

Opioids and gabapentinoids are used to treat acute and chronic pain and prescriptions of these medications have risen dramatically in recent years. Chronic pain is more common in people with lower socioeconomic status (SES)^1^ and the prescribing rates and adverse effects of these medications are higher in more socioeconomically disadvantaged groups.^2–4^ However, these medications have limited effectiveness in treating chronic primary pain (pain without a specific underlying cause).^5^ For example, increasing evidence exists that short-term use of opioids compared with non-opioid medications showed no difference for a range of outcomes (such as pain, sleep, and function) and the evidence of long-term effectiveness of opioids is very limited with growing evidence of dose-dependent increased risk of harms.^6^ Both opioids and gabapentinoids can lead to harmful short and long-term side effects, dependence, misuse and adverse events, including death.^2,4,7,8^

Guidance exists around prescribing these medications; for example, the US’s Centers for Disease Control and Prevention^9^ and the UK’s National Institute for Health and Care Excellence (NICE).^5^ The former recommends that opioids should not be considered first-line for chronic pain;^9^ NICE goes further advising against the initiation of opioids and gabapentinoids in patients with chronic primary pain in people aged ≥16 years^5^ with recent advice about withdrawal management.^10^ However, prescribing of these medications remains high. These guidelines advocate for person-centred care. This includes working collaboratively with patients, and tailoring the support that each patient requires to reach informed decisions about their health and wellbeing.^11^ Shared decision-making underpins person-centred care and involves healthcare professionals and patients working together to agree a plan; compromise and negotiation may occur.^12,13^ The patient’s preferences and the clinician’s expert knowledge are of equal value with the patient having the right to decide about their care as long as they are fully informed of their respective options.^12,13^ Therefore, agency, '*the ability to act or choose which action to take*’, is present.^14^ However, interventions requiring a high degree of self-agency are often found to widen health inequalities.^15^ Understanding how these elements are operationalised when deprescribing these medications, particularly in areas of high SES where need is often greater, as well as identifying which elements are viewed as key ingredients, are important aspects to establishing the key levers for change.

Established in 2020, the Deep End North East and North Cumbria (NENC) is a regional network supporting primary care professionals working in GP practices in the most socioeconomically deprived areas in North England.^16,17^ NENC Deep End practices face multiple challenges, including the mismatch between the volume and complexity of patients' needs versus the time and workforce available to provide the required support (compounded by severe staff recruitment and retention difficulties) as well as the healthcare system’s failure to recognise their complex challenges within the funding arrangements.^18^ Many Deep End practices have high prescription rates of opioids and gabapentinoids correlated with deprivation.^16^ In 2022, the Deep End NENC network piloted providing practices protected funded time to review and address their prescribing of these medications in high-risk patients. The opportunity was advertised by this network with six Deep End practices self-selecting. The list size of practices varied (ranging from 2500–11000 patients) and their location (for example, urban conurbation, small town, rural).^19^ The proportion of their list sizes who lived in the 15% most disadvantage neighbourhoods in England ranged from 46.6%–83.9% highlighting the high levels of deprivation within the communities served by these practices. The pilot included one session per week for 6 months to backfill GPs’ or allied professionals’ time to plan and deliver an intervention to reduce their opioid or gabapentinoid prescribing. Evaluation was built into the intervention’s design, with practices allocated an additional session per month to participate in the evaluation. Monthly peer-support meetings and additional training or support requests were offered. Each practice decided how to approach the work, including developing guidelines for some practices (Table 1).

**Table 1.** A summary of the key elements of the intervention (TAPER)

Element of the intervention	Considerations
**Design of the intervention**	
Primary care staff	Identified the staff who would be involved in the work Decided how to support professionals involved Worked with commissioners to design the evaluation such as the quantitative data collection template
Patients	Practices agreed which patients to prioritise Practices identified the agreed patients using the relevant primary care’s IT systems Practices agreed how the patients would be informed of the work. For example, some practices used a letter to invite the patients for a medication review or to inform the patient that a reduction would be occurring with an opportunity to discuss with the practice. Other practices used ad hoc phone calls
Medication reviews	Agreed how the medication reviews would be structured. This included deciding if implementing: thresholds for forcing a reduction particularly if on unsafe doses, or urine toxicology to assess if taking the prescribed medication (and help rule out divergence) and verify the presence of other medications (prescribed or not) that may indicate a higher risk Agreed mode of consultations to be offered (for example, in-person or over the telephone)
**Implementation of the intervention**	
Primary care staff	Undertook and documented the medication views Decided how to navigate difficulties such as patient non-attendance or refusal Participated in the evaluation
Patients	Agreed with patients the support they required to help taper their doses such as how often the patients would be reviewed and how

This study aimed to explore primary healthcare professionals' views and experiences of designing and implementing the TAPER (Deep End ProjecT: PrimAry care Professionals' Experiences of Reducing opioid and gabapentinoid prescribing in socioeconomically disadvantaged communities in the North East of England) intervention to reduce opioid and gabapentinoid prescribing in high-risk patients in Deep End practices serving socioeconomically disadvantaged communities. This included exploring the perceived acceptability, barriers, and facilitators of TAPER.

## Method

The sampling frame consisted of six self-selecting practices within the NENC Deep End network. A seventh Deep End practice, which had previously undertaken a similar project that influenced this pilot, was also included. Interpretive pragmatism was the epistemological stance incorporating a hybrid blended model, including ethnographic and thematic analysis approaches, to allow for multiple angles from which to ‘view’ the intervention.^20,21^ Data collection consisted of two components:

1. participant observation of healthcare professionals in the monthly peer-support meetings; and
2. semi-structured interviews with healthcare professionals at the relevant practices.

All participants were healthcare professionals with no patient involvement.

### Semi-structured interviews

Each participating practice was contacted via email outlining the study and inviting participation. Thereafter snowball sampling^20^ was undertaken whereby interviewees helped identify additional key informants in their respective practices. Interview questions were based on an iteratively developed topic guide (Supplementary Information), informed by normalisation process theory,^22,23^ which is a implementation theory considering how new ways of working are embedded and integrated into health care.^23^ An information sheet was emailed to perspective interviewees, and participants completed an online consent form. Interviewees were interviewed by CPC, using the online platform Teams or via telephone, lasting up to 1 hour. CPC (she/her) is a Public Health Registrar with a medical background and 18 months WTE public health research experience including qualitative. CPC organised the interviews and had no prior relationship with the participants apart from interaction in relevant meetings and email correspondence about the study. Participants knew the interviewer’s role, workplace, and aims of the research. Brief notes were taken; the interviews were recorded, transcribed verbatim, and anonymised, and a summary of the key findings, as well as policy and practice implications, were shared with the practices involved in the pilot.

### Participant observation

All social research is considered as forms of participant observation with involvement of participation in the social world.^24^ CPC attended each monthly 30-minute peer-support online session (through Teams) during the pilot (*n* = 4). All attendees were emailed an information sheet before the meetings, which was briefly discussed and shared again at the start of each meeting, with all attendees providing verbal consent. Participants were aware of CPC’s role and reason for attending. CPC’s role was as a minimally participating observer^25^ because CPC observed the meeting with camera on and muted, and only interjected infrequently to ask questions for clarification to achieve the study’s aims and objectives. Anonymised notes were taken using a template originally developed by Gibson.^26^ This template was adapted and included describing discussions and researchers’ reflections.

### Analysis

Interviews were coded inductively and analysed independently by two researchers (CPC, JP) with the participant observations notes being coded inductively by CPC considering the available resources and time. The approach to analysis was based on an essentialist thematic analysis with a sematic approach^27^ to identify key themes. The analysis was managed by NVivo software (version 14).

## Results

Sixteen primary care professionals were invited to participate from the seven general practices; 13 consented to participate from five practices. The practices that took part in the interviews are training practices. The breakdown was seven general practice doctors (practice partners or salaried; quote identifier: GP) and six pharmacists/pharmacy technicians (quote identifier: P), or allied primary healthcare professionals (quote identifier: A). Four peer-support online meetings were observed. Attendance at these meetings was optional and ranged from 3–7 professionals including the lead GP or lead pharmacist for the relevant practices and the Deep End programme manager. Each meeting was structured similarly: an update from each practice and discussion of difficulties with collective troubleshooting and sharing learning (including resources such as letter templates). Theoretical sufficiency was reached, and the four key themes discussed in this paper are:

1. Person-centred care with shared decision-making;
2. Risk;
3. Emotional tightrope; and
4. Culture change.

### Person-centred care with shared decision-making

Person-centred care was intertwined with shared decision-making when deprescribing these medications. This included pitching the communication in a format that made sense for that patient and adjusting to any additional needs they had. These elements helped promote agency and needed to be considered at every stage including how patients were informed of the deprescribing work.

*‘There are patients that are illiterate* [...] *patients that don't have access to the internet.* […] *we needed to give multiple options to people but also lay them out quite clearly.’* (P3)

Taking a person-centred approach appeared to be a way to facilitate patient buy-in and was considered a key requirement and skill when undertaking this work for patients to adhere to the agreed plan. Developing rapport was viewed as crucial; a balancing act between building a relationship with the patient versus assessing and mitigating risk (Supplementary Table, Quote 1). Some interviewees spoke about the importance of showing the patient that they cared by listening and being compassionate (Supplementary Table, Quote 2). These factors appeared to be regarded as facilitating the development of trust with the patient and acknowledging that the patients and their needs mattered to increase buy-in.

*‘In my experience,* [...]*, you've got to get the patient on board as soon as possible* [...] *I think there’s* [...] *an art and science to that, which I, I haven't quite mastered yet.’* (P1)

Shared decision-making was strived for and thought to be a key facilitator. It considers the clinician’s expertise (communicating the evidence, risk versus benefits, and treatment options).^28^ The way this was communicated varied and interestingly came with varying degrees of persuasion to help secure patient buy-in. Common important components included patient education about chronic pain and how its management had changed, as well as these medications' potential side effects, adverse effects, or consequences (including addiction, tolerance, dependency, and death). Interviewees spoke about healthcare professionals having partly created the situation whereby patients were prescribed inappropriate doses of these medications. Explaining this when communicating with the patients was considered useful to help the patients understand why deprescribing was now recommended.

‘*Often we've created this problem, it’s not the patient’s fault* […] *So I do try to explain that to patients and sometimes say look, we are reviewing these medications, we want to reduce people from it, it is our fault that you're on this medication, it’s probably not safe in the first place.’* (GP5)

Exploring the patient’s preferences, personal circumstances, goals and beliefs is important in shared decision-making.^28^ This was demonstrated by interviewees reviewing the patient notes before the consultation to help understand why the patient was on the medication(s) and considering how other elements in the patient’s life may affect their ability to reduce the medication(s) and exploring the patient’s views during the review.

*‘They* [patients] *need a conversation where it feels real to them, where you listen to their side of the story, listen to how they’ve ended up on it* [the medication]*, why they’re on it, what’s going on in their lives, and putting that into that context. That’s what needs to happen for them to see “Okay, fine this is how it might work.”’* (GP2)

Sometimes it was apparent it was not the right time to start tapering but agreeing to review in the future was a favourable alternative (Supplementary Table, Quote 3).

Being flexible, including discussing choices around how to undertake the reduction, was reported to be beneficial to help the patient feel they had some control as long as safety was not compromised. Patients’ preferences were explored to different degrees, for example:

- which medication to start with first (if on multiple);
- frequency of reviews and which mode (telephone or in-person) during tapering; and
- the dose to begin with.

During tapering, providing support for the patient, according to their needs, was important (Supplementary Table, Quote 4) as well as being flexible, where possible.

*‘And there has, since then, been some* [patients] *whereby they've reduced to a point, and they've said, "I think this is my lowest effective dose." And it’s about being a bit transparent and saying, "Okay, no problem. We'll pause you there. We might re-look at it."'* (P3)

Therefore, patient adherence was enhanced by person-centred care and shared decision-making. However, this approach was time-consuming and complex as patients had complex histories and lives. Discussed in both the interviews and peer-support meetings, having dedicated time for these reviews was considered crucial.

*‘It’s slow, it’s slow work, and you can’t speed it up because then you lose the faith of your patients and you've got to have them on board to be able to manage it.’* (P5; and Supplementary Table, Quote 5)

By facilitating this time and resource, this pilot was considered a good example of the benefits of being in the Deep End network amid the pressures that they are under.

### Risk

All the practices used risk to identify and prioritise which patients to invite. For all practices, ‘high risk’ was categorised as patients on high doses of opioids or gabapentinoids.

*‘We started by, I guess, essentially stratifying sort of groups of patients, so picking off very high-risk patients. So, they’d be patients on opiates, say, above 120 mg over a 24-hour period.’* (GP7)

Some practices included additional factors, such as timing of last medication review, age, comorbidities, previous history of drug or alcohol misuse and/or other concurrent high-risk medication(s) (Supplementary Table, Quote 6). This all contributed to identifying the patients who were at the greatest risk of suffering the largest consequences.

Navigating shared decision-making and agency can pose dilemmas for professionals working in this context. Shared decision-making was strived for but if not possible, forced reductions were taken by some practices. In these cases, risk was used to determine the thresholds for removing agency so that the patient had to reduce their medication doses (or even stop) either owing to life-threatening risks to that patient, or safety concerns that the patient was not taking the medication and diverting it to others (Supplementary Table, Quote 7). These difficult decisions were not taken lightly; despite not being a shared decision between clinician and patient, these decisions were shared between clinicians.

‘*And there were some patients who were generally on really unsafe combinations and doses of medicines who, we did, after several, you know, conversations and consultations with the patient, we did end up dose-reducing sort of against their will, but that was like a shared decision with myself, their GPs* […]' (P4)

The decisions taken by some practices that removed agency were not always owing to risk but around enabling the agreed goal. These decisions included requirement to opt-out of the reviews, forced reductions, or during tapering patients could remain on a certain dose for longer but not increase. Some of the success in achieving a prescribing reduction was attributed to these stricter approaches (Supplementary Table, Quote 8). Conversely, the decision by other practices not to push too hard or remove agency by forcing the reduction were owing to concerns that it would not work (for example, the patient was likely to find another way of getting the medication), that it went against shared decision-making principles, or the potential impact on that patient’s mental health.

### Emotional tightrope

This work evoked a range of both negative and positive emotions in staff and patients, which was apparent in both the interviews and peer-support meetings. Firstly, uncertainty about how best to approach the work existed for the healthcare professionals, as even though the guidelines had changed about prescribing these medications, limited guidance existed about how to go about the reductions or what to do if the patients did not engage or refuse. The peer-support meetings provided opportunities to discuss ways of troubleshooting these challenges or acknowledging how difficult this type of work could be. Some participants in the peer-support meetings appeared to be more willing to speak candidly about the challenges they had encountered than others. This may be owing to a fine balance between seeking support versus being concerned about being judged. There was also uncertainty about how the patients would react to the work. These elements led to apprehension for staff.

*‘What do you do if they refuse to even consider any kind of withdrawal or switch? I guess that’s kind of individual decision for everyone involved, but yes, how far do you push these things?’* (GP5)

When patients engaged, the consultations were often difficult and stressful owing to patients’ emotional response and resistance.

*‘And emotional energy, because they’re* [the consultations] *really draining, some of them.’* (GP2)

Interviewees reported instances of angry, manipulative and/or threatening behaviour by patients (Supplementary Table, Quote 9).

Interestingly, participant observations during the peer-support meetings highlighted an aspect that was not prominent in the interviews, which was a small number of practices found it more challenging to manage the younger population owing to resistance to change. Overall, the patients' negative reactions and resistance appeared to stem from underlying concerns that reducing the dose(s) would negatively impact their quality of life (Supplementary Table, Quote 10)*,* which was fuelled by:

- not understanding why taking these medications was now a concern when they had been taking them for a long time, started by a healthcare professional, with seemingly often no perceived side effects. This was sometimes despite still being in pain; and
- having negative experiences trying to manage their pain or reduce previously (for example, rebound pain or withdrawal).

*‘What I am struggling with are the patients that have been on long term, high dose, or they’re involved in, you know, secondary care, pain clinics, or musculoskeletal services or anything like that, who are on them, and they’re kind of questioning, “Well, why are we changing now? I’ve got no problem, why should I change?"’* (P2)

Shared decision-making involves the healthcare professional exploring the treatment options with the patient; however, interviewees reported frustrations owing to a perceived lack of alternatives as they were either:

- contraindicated or the threshold for referral was not met;
- not perceived as suitable or acceptable for their patient demographic;
- had long-waiting lists; or
- had already been tried and not worked.

*‘I think in a struggling working class area, you know, where you have people* [....] *who are taking painkillers for joint pain, if you turn around and say, “I'm going to take your painkillers off you, but I'm going to send you to Talking Therapy* [psychological therapies] *instead,” that can be a very difficult thing to sell. Even though we have good evidence for it.'* (P1)

Despite being difficult work, the healthcare professionals reported that it was sometimes easier and more rewarding than expected.

*'I think there’s always this feeling among doctors, you know, that trying to reduce them will always be met with a hostile and defensive response, but actually, there are people who are quite keen and willing to reduce and feel better when they get on less medication, so it’s not, it’s not all a battle.’* (GP1)

### Culture change

The ultimate goal was the need to change the culture to ensure success and sustainability: patients should not be on inappropriate doses of these medications for inappropriate reasons. This includes both new prescribing and deprescribing of these medications and encompasses not only primary care but also the wider healthcare system. Some participants were even explicit about this in both the interviews and peer-support meetings.

*‘I think it’s about trying to instil a culture around opiate prescribing because I feel like if we're chipping away at people’s MST and Zomorph at one end, and then other members of staff are starting people on Zomorph and MST for inappropriate indications at the other end, then it’s going to be never-ending.’* (P1)

Demedicalising pain was viewed as important to facilitate this culture change. This included helping patients learn to live well with pain but acknowledging that chronic pain is complex and hard to cope with (Supplementary Table, Quote 11).

*‘I spend a lot of my life speaking to people of 70, 80, or 90* [years old] *saying, “Why isn’t my body very good?” You have to say, “Well your body is getting older, it’s not going to work so well. This is normal. To have some pain is normal, you don’t have to have a tablet to take it away."’ (*GP3)

Limiting the number of these medications being started in the first place was viewed as imperative. If having to start them, recommendations were to use low doses initially and manage patients’ expectations at the outset that these medications were only short term with ongoing review. This culture change may help reduce or even remove the expectations from patients that healthcare professionals will cure their chronic pain and that alternatives should or would be provided.

*'The other response that we get commonly as well is, “Okay, so if you stop my morphine, what am I going to get instead?”* [...] *the patients have a sort of expectation that, you know, this pain that they have every day, if it can't be treated with morphine, then it’s going to have to be treated by something else.’* (P1)

This may help alleviate the pressure that healthcare professionals potentially feel about providing another pharmacological treatment (Supplementary Table, Quote 12).

## Discussion

### Summary

This study captures how primary care health professionals view and experience designing and implementing an intervention to reduce opioid and gabapentinoid prescribing in high-risk patients in Deep End practices. It captures rich and candid data of how professionals felt about designing and delivering this work as well as the barriers and facilitators. The four main themes discussed were as follows: person-centred care with shared decision-making; risk; emotional tightrope; and culture change. The findings were used to develop recommendations for commissioners and primary care settings, including practices covering socioeconomically deprived areas (Table 2).

A key concept is changing the culture both in primary care and the wider healthcare system. For example, moving away from the medical model, which has been reported to be used for social problems in areas of deprivation whereby distress is medicalised, manifesting in patients being prescribed pain medication inappropriately,^18^ potentially leading to patients having unrealistic expectations of what health care should offer.

Deprescribing is time-consuming, can be emotionally draining, and includes difficult decisions for professionals, particularly when patients refuse to reduce in the context of either high-risk doses, high-risk combinations, or have comorbidities that substantially increase their risk. Guidance from, the UK’s General Medical Council and NICE, exists to help practitioners with difficult prescribing decisions. For example, doctors should *'provide effective treatment based on the best available evidence'*.^29^ When professionals are faced with requests to provide patients with medication unlikely to be beneficial, the professional should explore the patient’s reasons, understanding, and expectations.^29^ Ideally, a shared decision should be sought but if this is not possible, and the professional considers that this medication does not serve the patient’s '*needs'*, the medication should not be provided with an explanation and exploration of other options, including a second opinion.^10,29^ However, '*needs*' are not defined leading to ambiguity, especially when the different types of needs, as defined by Bradshaw, can vary greatly.^30^ For example, normative needs could be argued as being what the clinician deems the patient *requires* versus the patient’s felt needs, which centre around what the patient *wants* to feel better. The latter is particularly challenging in the context of drug dependency and possible addiction.

**Table 2.** Key recommendations for practices considering undertaking deprescribing of these medications

Key recommendation	Rationale with further information
**Designing and implementing the intervention**	
Funding dedicated time to enable deprescribing	Deprescribing takes time to design and implement, therefore funding dedicated time enables prioritisation of the work in the context of potentially conflicting pressures in primary care
Each practice should agree internally how they will select, identify, and notify the relevant patients	To facilitate a systematic approach, enabling prioritisation of patients with the highest risk. Invitation for the medication reviews may include letters (with opt-out) or ad hoc phone calls and should be easy to understand with needs of the individual patient being considered. This is particularly important if patients are, for example, visually impaired, unable to read or need translation
Each practice should agree internally how they will approach the medication reviews including steps to undertake for non-engagement or patient refusal to reduce	To develop a consistent approach including dealing with these difficult decisions. GMC and NICE guidance can assist with the decision-making. This step should also include identification of a team in the practice to undertake the work (such as GPs and pharmacists or pharmacist technicians working together)
Support and training for the health professionals undertaking the deprescribing work should be made available	To help staff manage complex pain management as well as the emotional aspects of this work. Teamwork, debrief sessions, or supervision and learning from others or training sessions were viewed to work well
**Structure of the content of the medication reviews**	
Being patient-centred and building rapport is important	To facilitate patient buy-in. Key aspects included taking the time to listen to the patient, including why they are on these medications, how they feel about these medications, and other factors in their life that may affect engagement with tapering
Communicate that the guidelines have changed is important alongside the evidence-base for managing complex pain	To help patient understand why deprescribing is now recommended. This should be done in an easy-to-understand way; analogies were viewed to work well
Offer choices to the patient about the process of tapering where possible	To enable the patient some control over the tapering process to facilitate patient buy-in and successful negotiation. Options include which medication to reduce first (if on multiple medications), how frequently the reviews should occur, by what mode (telephone or in-person), and the initial dose to reduce by
Offering support tailored to the patient during tapering	To provide a safety net for patients during tapering. This includes regular check-ins with the patient at a suitable time and mode

GMC = General Medical Council. NICE = National Institute for Health and Care Excellence

### Strengths and limitations

To our knowledge, this is the only UK study to explore primary care professionals' views and experiences of deprescribing both opioids and gabapentinoids in Deep End practices that cover significant deprivation. Several practices were involved from across the region at varying stages of their deprescribing journey with interviewees from different professional backgrounds adding to the findings' richness. Data were collected not only from interviews but also from observations at the peer-support meeting enabling integration of the findings. By way of positionality and self-reflexivity, the lead researcher is a public health doctor who has worked on public health projects related to opioid harm reduction, but not in general practice. This may have affected the interview approach and interpretation, which was minimised by using a topic guide informed by an established framework, second coding, and theme discussion. Additionally, the researcher’s presence at the peer-support meetings for the participant observation may have influenced the dynamic, which was minimised by the researcher only having minimal involvement in these meetings. As interviewees were only interviewed once during the process, their perceptions may have changed during the pilot. However, the findings and recommendations were shared with the pilot practices and reported to be an accurate reflection. Patients were not involved in the pilot’s design or evaluation, which should be considered in future work. This involvement would strengthen the role of shared decision-making in guidance or intervention development with previous research finding that patients involved in opioid deprescribing wanted to be more involved in the decision-making process.^31^

### Comparison with existing literature

Previous literature has explored patients' perspectives of effective pain management,^32^ deprescribing of these medications,^31,33–36^ or on harm reduction models (for example, opioid agonists treatment or opioid antagonists in opioid overdose such as naloxone).^37,38^ Where professionals' perspectives were sought, opioid overdose education,^39^ attitudes regarding management of chronic pain^40^ (including chronic joint pain),^41^ and opioid prescribing^42–45^ dominates. Where deprescribing was explored, the North American-based studies cited emotional burden on primary care professionals as these types of conversations were described as time-consuming, difficult, and exhausting with patients resisting.^34,46^ This was seen in our study, but it was not uncommon that some consultations were positive with patients willing to taper. Taking an individualised supported person-centred approach was highlighted as a key facilitator, which is similar to our findings.^31,34,46,47^ A UK-based qualitative study, evaluating the provision of feedback to practices about their opioid prescribing, concentrated on how practices received and responded to the feedback.^48^ This study also found that enabling practices the flexibility to decide what actions should be taken by each practice was a key facilitator. However, the importance of culture change in these studies was not highlighted as a key theme, which is different to our study.

### Implications for research and practice

Table 2 outlines our key recommendations for commissioners and primary care practitioners. Time and flexibility were considered key to develop and implement the intervention and enable person-centred care with shared decision-making. This approach should be considered by commissioners and GP practices wanting to reduce the prescribing of these medications. Further guidance clarification about how to navigate patient refusal or non-engagement is necessary. Acknowledging the emotional toll with the provision of support for patients and healthcare professionals is required. This includes the importance of training in complex pain management, prescribing and deprescribing of these medications, and how to navigate patient refusal or non-engagement. This should consider the key cognitive, behavioural, and environmental factors required to improve patients' self-efficacy to engage with the deprescribing process. with inclusion of psychosocial guidance and behavioural change techniques, to facilitate a person -centred approach to overcome the relevant barriers for each individual..^31,47^ Research into healthcare system-wide change, particularly around the interface between primary and secondary care with person-centred deprescribing of these medications in areas of deprivation, is warranted. This includes navigating the challenges that come with striking a balance between shared decision-making and agency.

## Data

The data supporting the findings of this research are available from the corresponding author upon reasonable request.
